# Complete Genome Sequence of Proteus mirabilis Phage Mydo

**DOI:** 10.1128/MRA.01312-19

**Published:** 2019-11-21

**Authors:** Benjamin T. Jones, Lauren Lessor, Chandler O’Leary, Jason Gill, Mei Liu

**Affiliations:** a Center for Phage Technology, Texas A&M University, College Station, Texas, USA; Queens College

## Abstract

Proteus mirabilis is a pathogen that has been linked to nosocomial infections. Studies on phages infecting P. mirabilis may provide therapeutics for infections caused by antibiotic-resistant strains of this pathogen. Here, we announce the complete genome sequence of a P. mirabilis myophage, Mydo, which is distantly related to Escherichia coli phage rv5.

## ANNOUNCEMENT

Proteus mirabilis is a Gram-negative enteric pathogen that is linked to a variety of hospital-acquired illnesses (1). It is intrinsically resistant to nitrofurantoin and tetracycline (1) and has been reported to have developed resistance to extended-spectrum cephalosporins and coresistance to other antibiotics due to the production of β-lactamases (2, 3). The study of phages infecting P. mirabilis may lead to alternative treatments for these antibiotic-resistant strains.

Phage Mydo was isolated from a wastewater sample collected from College Station, TX, in 2013 using a Proteus mirabilis isolate as the host. Host bacteria were cultured on nutrient broth or agar (Difco) at 37°C with aeration. Phages were cultured and propagated using the soft agar overlay method (4). It was identified as a myophage using negative-stain transmission electron microscopy performed at the Texas A&M University Microscopy and Imaging Center, as described previously (5). Phage genomic DNA was prepared using a modified Promega Wizard DNA cleanup kit protocol, as described previously (5). Pooled indexed DNA libraries were prepared using the Illumina TruSeq Nano low-throughput (LT) kit, and the sequence was obtained from the Illumina MiSeq platform using the MiSeq V2 500-cycle reagent kit, following the manufacturer’s instructions, producing 1,112,580 paired-end reads for the index containing the phage Mydo genome. FastQC 0.11.5 (https://www.bioinformatics.babraham.ac.uk/projects/fastqc/) was used to quality control the reads. The reads were trimmed with FASTX-Toolkit 0.0.14 (http://hannonlab.cshl.edu/fastx_toolkit/download.html) before being assembled using SPAdes 3.5.0 (6). Contig completion was confirmed by PCR using primers (5′-GGTGTCTGGTACGTTGGTTC-3′ and 5′-TGTGTGTGACAACGTACCTG-3′) facing off the ends of the assembled contig and Sanger sequencing of the resulting product, with the contig sequence manually corrected to match the resulting Sanger sequencing read. Glimmer 3.0 (7) and MetaGeneAnnotator 1.0 (8) were used to predict protein-coding genes with manual verification, and tRNA genes were predicted with ARAGORN 2.36 (9). Rho-independent terminators were identified via TransTermHP v2.09 (http://transterm.cbcb.umd.edu/). Sequence similarity searches were performed by BLASTp 2.2.28 (10) with a maximum expectation cutoff of 0.001 against the NCBI nonredundant (nr), UniProt Swiss-Prot (11), and TrEMBL databases. InterProScan 5.15-54.0 (12), LipoP (13), and TMHMM v2.0 (14) were used to predict protein function. All analyses were conducted at default settings via the CPT Galaxy (15) and Web Apollo (16) interfaces (https://cpt.tamu.edu/galaxy-pub).

Phage Mydo was assembled at 81-fold coverage to a complete genome of 145,127 bp. Mydo has a G+C content of 45%, which is higher than that of its host (39%) (17). At both the nucleotide level and protein level determined by BLAST against the NCBI nr/nucleotide database (E value, <0.001), Mydo is closely related to Klebsiella phages vB_KpnM_BIS47 (GenBank accession number KY652726) and vB_KpnM_KB57 (GenBank accession number KT934943). Mydo shares 86% and 83% DNA similarity and 230 and 223 proteins (out of 264 total predicted proteins in Mydo) with phage vB_KpnM_BIS47 and phage vB_KpnM_KB57, respectively. With 77 shared proteins (determined via BLASTp; E value, <0.001), phage Mydo is also distantly related to Escherichia coli phage rv5 (GenBank accession number NC_011041) (18), placing it within a cluster of large, virulent myophages that infect Gram-negative hosts.

### Data availability.

The genome sequence of phage Mydo was submitted to GenBank as accession number MK024806. The associated BioProject, SRA, and BioSample accession numbers are PRJNA222858, SRR8771451, and SAMN11234226, respectively.
